# Coupling and Coordinating Relationship between Tourism Economy and Ecological Environment—A Case Study of Nagasaki Prefecture, Japan

**DOI:** 10.3390/ijerph182312818

**Published:** 2021-12-05

**Authors:** Yiming Liu, Sunhee Suk

**Affiliations:** Graduate School of Fisheries and Environmental Sciences, Nagasaki University, Nagasaki 852-8521, Japan; bb53421008@ms.nagasaki-u.ac.jp

**Keywords:** PSR model, tourism development, ecological environment, coupled and coordinated development

## Abstract

The tourism environment is the basis of sustainable development in the tourism economy. Exploring the coupling relationship between tourism economy and ecological environment systems can promote not only ecology-based tourism, but also contribute to the sustainable development of tourism economy. Based on data from Nagasaki Prefecture, Japan, from 2010–2019, this paper aims to introduce an indicator system and develop an integrated approach to assess the coupling and coordination between the tourism economy and the environment. The indicator system consists of two levels, six aspects, and eighteen indicators, based on entropy method. A Pressure-State-Response (PSR) model framework of the coupling and coordination mechanism of tourism economy and ecological environment was constructed based on the development status of Nagasaki Prefecture in Japan. Then, the degree of coupled coordination of its tourism economy and ecological environment is evaluated, providing a comprehensive evaluation index of the system. In conclusion, suggestions for promoting the sustainable development of tourism and environment in Nagasaki Prefecture, Japan, are proposed. The purpose of this research is to reveal dynamic trends that exist between the coupled development of tourism economy and the ecological environment. A further aim of this paper is to provide a reference for macro policy formulation in small and medium-sized cities regarding the sustainable development of the tourism economic system and ecological environment system.

## 1. Introduction

As a resource and environment-dependent industry, tourism is inextricably linked with the environment. On the one hand, a healthy ecological environment is the guarantee and source of development of the tourism economy, which in turn will promote further improvement of the ecological environment. On the other hand, the excessive pursuit of economic benefits will lead to a deteriorated ecological environment, which in turn will affect the sustainability of the economic development of tourism. Therefore, the study of coupled and coordinated development of tourism and environmental ecology has become a hotspot for academic research. This has important theoretical and guiding values.

Researchers have studied the relationship between the environment and tourism as far back as 1920 [1]. In this initial phase, scholars began to study the ecological impacts of tourism activities, and most studies comprised general observations and qualitative descriptions [2,3]. The development of tourism has since led to adverse effects on ecology and the environment, thus researchers have begun to focus on the impacts of tourism on the ecological environment [4,5,6]. Many researchers have focused on the negative impacts of tourism on the water [7,8] and air environments [9,10]. In the 21st century, as research continues to deepen, research on the relationship between ecological environment and tourism has gradually become a research hotspot, and research into the relationships between tourism and the ecological environment have become more specific and diverse, especially in the issues of tourism capacity and tourism carrying capacity [11,12], tourism ecological safety [13,14], tourism resources development and ecological environment protection [15,16] and the impact of climate warming on tourism development [17,18]. Researchers have introduced many models and quantitative methods, which have inspired research into the harmonious development of tourism and the environment. In this research field, considering such variables as part of a system, a form of modelling called the Coupling Coordination Degree Model (CCDM) can be used, which reflects the tendency of a system to evolve towards an ordered state [19]. CCDM can be used to trigger benign interactions between subsystems to ensure they evolve in harmony [20]; therefore, such modelling has been widely used in the research of related social and economic fields—in particular, to study the coupling of economic development and ecological environment [21,22], population and economic development [23,24], water resources and economic development [25,26], and urbanization and ecological environment [27,28]. Among them, most researchers have conducted research on tourism and the environment [29,30], as well as the coupling and coordination with tourism from the perspective of tourism traffic [31,32], urbanization [33,34] and air environment [35,36].

In summary, the basis of research methods on the relationship between the ecological environment and the tourism economy has gradually changed from qualitative to quantitative, and coupled coordination models have been more widely applied [37,38,39]. However, existing studies are mainly limited to large cities at mature stages of tourism development, as well as whole countries, while small and medium-sized cities are neglected as the basic units of interaction between the ecological environment and tourism economy [40,41,42]. Therefore, this paper takes Nagasaki Prefecture in Japan as the research object, and based on the Pressure-State-Response (PSR) model framework for researching issues related to the environment, resources and sustainability, introduces a comprehensive index system reflecting the coupling relationship between the ecological environment and the tourism economy. Using the Coupling Coordination Degree Model (CCDM), the degree of coupling and coupling coordination of the tourism economy and ecological environment of Nagasaki Prefecture are studied for the years 2010, 2013, 2016 and 2019. This research had two aims; one was to reveal dynamic trends in the coupled development of tourism economy and ecological environment, and the other was to provide a reference for formulating macro policy on sustainable development of the tourism economic system and ecological environment system in the environment for small- and medium-sized cities.

## 2. Study Area

**Figure 1:**
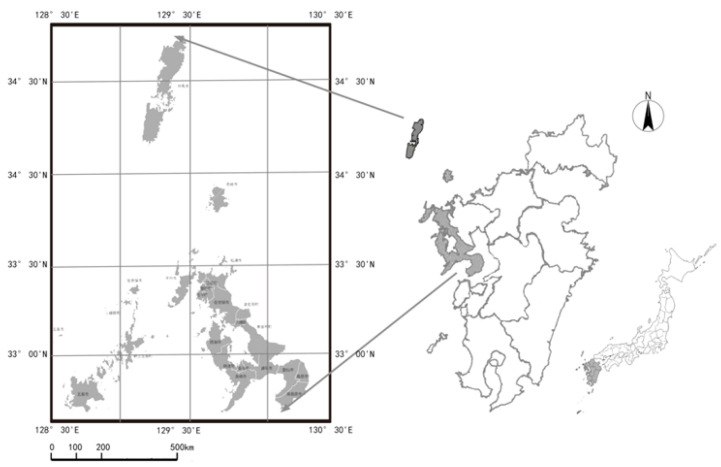
Location of Nagasaki Prefecture (Source: the authors).

The study area was Nagasaki Prefecture, located in the westernmost part of Kyushu Island, at the southernmost tip of mainland Japan. It is also the closest point in Japan to the Korean Peninsula, mainland China and, therefore, mainland Asia. The prefecture has 13 cities, the prefectural capital of which is Nagasaki City. The climate is typically maritime, with an average annual temperature of 18 °C and annual precipitation of 1464 mm. The prefecture also has the second-longest coastline in Japan at 4203 km, and is home to the largest fishery resources in Japan and a variety of fish species. See Figure 1 for the location.

## 3. Materials and Methods

### 3.1. Assessment Index System

The evaluation indicator system is the basis for researching the degree of coupling and coordination between the ecological environment and the tourism economy. For this paper, a systematic literature review was conducted on ecological environment and tourism economy. Expert group discussions were used to determine the PSR model to construct an evaluation index system for the tourism economic system and ecological environment system in Nagasaki Prefecture, Japan. The PSR model was developed by the Organization for Economic Co-operation and Development (OECD) and the United Nations Environment Program (UNEP) to study issues related to the environment, resources, and sustainability [43]. The model framework is based on a very clear causal relationship [44]. On the one hand, human activities have caused a certain amount of pressure on the ecological environment, thus causing changes in the ecological environment. On the other hand, human society should respond to environmental changes by restoring the quality of the ecological environment or preventing further environmental degradation. At present, the PSR model has been widely recognized and applied in the fields of environment [45,46], ecology [47,48], and ecological security [49,50].

Therefore, this paper analyses the actual conditions of the tourism economy and ecological environment of Nagasaki Prefecture based on each module in the PSR model as well as the principle that the indicators need to be representative, scientific and objective. After conducting many expert discussions, 19 evaluation indicators used frequently by researchers in recent years were selected. A PSR model indicator system for the ecological environment system and tourism economic development system of Nagasaki Prefecture in Japan was then constructed (Table 1) [51,52,53]. Of the indicators for the tourism economic system, nine indicators were selected from the three dimensions of ecological resources, ecological environment and development potential to comprehensively evaluate the development level of the tourism economy. In accordance with the PSR model, for the ecological environment system, 10 indicators were selected in three dimensions to comprehensively evaluate the development level of the ecological environment.

### 3.2. Data Resourses

This paper used panel data (2010, 2013, 2016, and 2019) from Nagasaki Prefecture. Tourism economic data was obtained from the Nagasaki Prefectural Tourism Trends Survey and Nagasaki Prefectural Tourism Statistics and ecological environment data were derived from the Ministry of the Environment’s official website, the e-Stat statistics website and the website of the National Institute for Environmental Studies of Japan.

### 3.3. Data Standardization

The tourism economy and eco-environmental system is comprised of several index layers, and the dimensions of each index differ, as do the directions of the forces. Therefore, the values of the indexes need to be standardized to enable comprehensive evaluation [54]. Assuming that the *m*-th index value of the *n*-th year in a certain place is *x*_nm_, the maximum value of index j is *x*_max_ and the minimum value is *x*_min_. According to the positive and negative properties of the index, the normalized value of *x*_nm_ can be obtained.

Positive index (larger value for a useful parameter):

(1) $$x\prime_{\mathrm{nm}}\;=\;\frac{x_{\mathrm{nm}}-x_{\min}}{x_{\max}-x_{\min}}$$

Negative index (smaller value for a useful parameter):

(2) $$x\prime_{\mathrm{nm}}\;=\;\frac{x_{\max}-x_{\mathrm{nm}}}{x_{\max}-x_{\min}}$$

### 3.4. Index Weight Calculation

Since the entropy method is more objective than the subjective analysis method, this study uses the entropy method to determine the weight of the index and avoid the influence of subjective factors. Following the calculation process of the entropy method, calculations are made, in order, of the normalized index proportion *S_nm_* of index *m* (Equation (3)), the entropy value *h_m_* of index *m* (Equation (4)), and the difference coefficient *α_m_* of index *m* (Equation (5)). Then, the weight of the indicator *w_m_* (Equation (6)) is determined.

(3) $$S_{nm}\;=\;x\prime_{\mathrm{nm}}/\sum_{n\;=\;1}^{p}x_{\mathrm{nm}}$$

(4) $$h_{m}\;=\;-\frac{1}{\ln p}\sum_{n\;=\;1}^{p}x_{\mathrm{nm}}$$

(5) $$\alpha_{m}\;=\;1-h_{m}$$

(6) $$w_{m}\;=\;\alpha_{m}/\sum_{m\;=\;1}^{q}\alpha_{m}$$

### 3.5. Sub-System Development Index Calculation

This research uses the weighting method to calculate the development level index *P_n_* of a certain subsystem in a certain place in the i year.

(7) $$P_{n}\;=\;\sum_{m\;=\;1}^{q}w_{m}x\prime_{mn}$$

### 3.6. Development of the CCD Model

As the coupling degree can only reflect the degree of interaction between the economic system and the ecological environment system and cannot effectively measure the synergistic effect of their overall development, a model to measure the degree of coupling coordination between the two based on the coupling degree model was developed for this study. The model developed can judge the level of coordinated development of tourism economy and ecological environment more scientifically. The calculation formula is:

(8) $$D(P_{\mathrm{TE}},P_{\mathrm{EE}})\;=\;\sqrt{C\times T}$$

(9) $$C\;=\;\sqrt{\frac{P_{\mathrm{TE}}\times P_{\mathrm{EE}}}{{\left(P_{\mathrm{TE}}+P_{\mathrm{EE}}\right)}^{2}}}$$

(10) $$T\;=\;\alpha P_{\mathrm{TE}}+\beta P_{\mathrm{EE}}$$

where D denotes degree of coupling coordination; C is the coupling degree of the two systems; T is the comprehensive coordination index of the two systems; P_TE_ and P_EE_ are the comprehensive evaluation indexes of the tourism economic system and the ecological environment system, respectively; and *α* and *β* are undetermined coefficients. Since the tourism economic system and the ecological environment system are of equal importance, values of *α* and *β* of 0.5 were used in the actual calculations of this research.

Due to the different magnitudes and dimensions of C and T, they were standardized using the following formula:

(11) $$C\prime \;=\;\frac{C-C_{\min}}{C_{\max}-C_{\min}}\times 0.9+0.1$$

(12) $$T\prime \;=\;\frac{T-T_{\min}}{T_{\max}-T_{\min}}\times 0.9+0.1$$

where C and T are the standardized coupling degree and comprehensive development index; C and T are the original values of the coupling degree and the comprehensive development index; C_max_, C_min_, T_max_, T_min_ are the maximum and minimum values of coupling degree and comprehensive development index in the original values.

### 3.7. Grade Division of the Coordinated Development

The values resulting from the coupling coordination D for the two systems for 2010–2019 were found to be mainly in the smaller range of 0.3–0.6. Therefore, in order to more clearly distinguish the coupling and coordination relationship between tourism economy and ecological environment, this research uses the tenth method of coupling coordination degree to classify the coordinated development level [55]. The specific classification criteria are shown in Table 2.

## 4. Results and Discussion

### 4.1. Characteristics of Comprehensive Development of Tourism Economy and Ecological Environment

**Figure 2:**
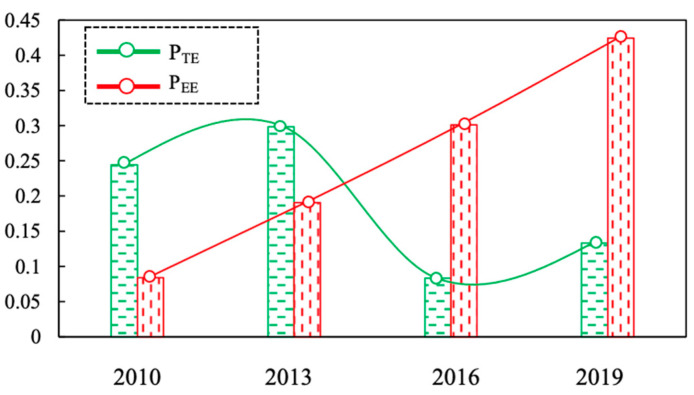
Comprehensive development index of tourism economy and eco-environment.

**Figure 3:**
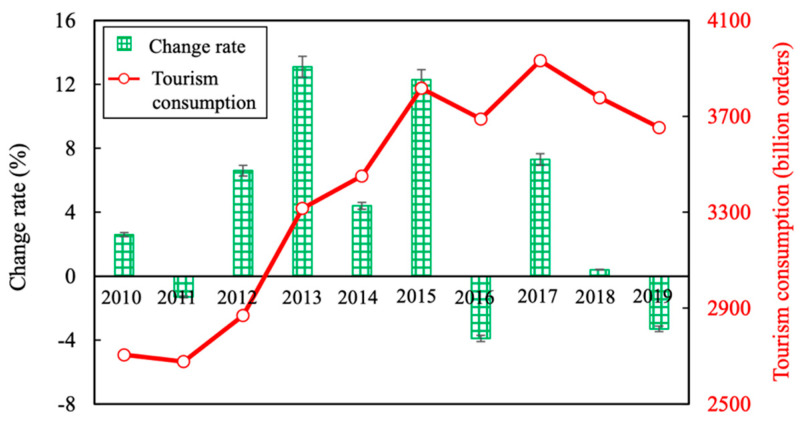
Tourism consumption and tourism consumption growth rate in Nagasaki Prefecture.

Through use of the coupling degree model and the coupling coordination degree model, this research obtained the comprehensive development index P_TE_ and P_EE_, the coupling degree C, the integrated evaluation index T of the tourism economic system and ecological environment system, and the coupling coordination degree D for Nagasaki Prefecture for the period from 2010 to 2019. Table 3 and Figure 2 below show the specific results obtained from this study, which are based on the classification in Table 2:

(1) From the perspective of the comprehensive development index of the ecological environment, the ecological environment development index of Nagasaki Prefecture from 2010 to 2019 showed a fluctuating decline (0.245 to 0.133). In particular, 2013–2016 showed a sharp downward trend, after which a slight increase occurred. This indicates that although the ecological status of Nagasaki Prefecture is good and the ecological pressure is decreasing year by year, the response to the ecological environment is insufficient. Naturally, the waste treatment rate, sewage treatment rate and waste recycling rate in Nagasaki Prefecture have all been greatly reduced, which has suppressed the overall rise in environmental damage.

(2) From the perspective of the comprehensive tourism economic development index, the tourism economic development index for Nagasaki Prefecture from 2010 to 2019 exhibited an increasing trend (from 0.084 to 0.424), with the rate of development increasing year by year. This is mainly due to the following three reasons:

In 2013, the prefecture was certified as one of the World’s top three new night scenes, and night tourism experienced rapid growth (Figure 3).

In 2015, it was registered as a World Heritage Site for its ‘Industrial Revolution Heritage of Meiji Japan Steelmaking/Steelmaking, Shipbuilding and Coal Industry’, which increased its appeal as a tourist destination.

Then, in 2018, the prefecture received recognition from Hidden Christians UNESCO World Heritage’ for sites within the prefecture, which greatly increased the number of cruise ship tourists and visitors to surrounding facilities.

(3) From an overall perspective, Nagasaki Prefecture’s PTE was less than its PEE from 2010 to 2013, meaning that the comprehensive development index of tourism economy was lower than the comprehensive development index of ecological environment at this stage, as it relates to the lag in developmental stage of the tourism industry. This shows that, on the one hand, tourism activities are still within a controllable range, in terms of their effect on the ecological environment. On the other hand, there is also much room for progress in the development of tourism. Then, from 2016 to 2019, the PTE of Nagasaki Prefecture was greater than the PEE, or, the tourism economic development index exceeded the ecological environment development index. This shows that while the level of tourism economic development rapidly improved from its origins, the level of ecological environment followed a significant downward trend, reflecting that the development of tourism in the prefecture has led to coercive pressure on the ecological environment.

### 4.2. Characteristics of the Coordinated Evolution of Tourism Economy and Ecological Environment Coupling

The coupling degree and coordinated development degree of tourism economy and ecological environment system of Nagasaki Prefecture from 2010 to 2019 can be calculated based on the data obtained in Table 3 and Formulas (8)–(10). From the results in Figure 3 and the metric based on the coupling degree (Table 4), it can be seen that the coupling degree index of the tourism economy and eco-environment system of Nagasaki Prefecture developed steadily since 2010, with the coordination degree index showing an overall fluctuating upward trend. The degree of coupling between tourism economy and ecological environment in Nagasaki Prefecture was mainly in the antagonistic stage, i.e., C was between 0.3–0.5.

The above can be interpreted to mean that the coordination effect of various factors between the tourism economy and ecological environment system of Nagasaki Prefecture was not obvious enough. The coupling between the tourism economy and the ecological environment was at a low level, and the negative effects of the growing tourism industry on the ecological system were becoming apparent. This was mainly due to the immature development of the tourism industry in Nagasaki Prefecture, pointing to the fact that the structure of the tourism industry needs to be optimized. In other words, this means that if tourism in Nagasaki Prefecture continues to develop at such a rapid pace, it is bound to have negative impacts on the ecological environment. Over the entire study period, the coupling between tourism and the ecological environment in Nagasaki Prefecture was in a state of growth (0.164 to 0.279), which indicates that the mutual influence and interaction between the two was gradually increasing. This shows not only that ecological conservation in Nagasaki Prefecture drives tourism development, but that tourism development promotes ecological conservation.

### 4.3. Coupling and Coordinated Development

From Figure 3 and the coordination degree measurement standard (Table 2), it can be seen that from 2010 to 2019, the coupling coordination degree of the tourism industry and the ecological environment in Nagasaki Prefecture exhibited a fluctuating upward trend (0.268 to 0.345), from a moderate disorder to a light disorder. The coupling coordination degree was lowest in 2010, at only 0.268, i.e., moderate disorder. This was due to the lowest comprehensive development index of the tourism economic system of Nagasaki Prefecture in 2010, i.e., that the development of tourism was in its infancy. In addition, PEE was much larger than PTE, and the comprehensive development level of the tourism economic system and the ecological environment system itself was relatively low, resulting in poor coupling coordination. There was a slight downward trend in the coupling degree from 2013 to 2016, which was mainly due to the impact of the significant decrease in PEE. From 2016 to 2019, the coupling coordination degree of the tourism economy and the ecological environment system of the prefecture continuously improved, and rose to its highest level in 2019 (during the research period) in the development stage of mild imbalance.

The above shows that although Nagasaki Prefecture adopted a series of measures to ensure development took a good path, the coordination between the tourism economic system and the ecological environment system of the prefecture was not high. With the emergence of other new forms of tourism such as ecotourism, health tourism, and green tourism, the comprehensive development level of the tourism economy and ecological environment of Nagasaki Prefecture has continuously improved, which increased the coupling and coordination between the tourism economic system and the ecological environment system in Nagasaki Prefecture.

## 5. Suggestions

(1) The level of tourism development in Nagasaki Prefecture has been growing year on year, and its infrastructure development for tourism, as well as capital investment and policies directed at tourism are relatively stable. Therefore, in the future, the prefecture’s tourism industry should focus on optimizing its structural makeup, and embrace land use planning, environmental protection planning, urban and rural development planning, forest land protection planning, and cultural relics protection planning, as well as strengthen the protection and management of tourism resources. In addition, the economic sustainability of tourism is a central issue for Nagasaki Prefecture. The tourism management department can attach tourism experience management to the tourism experience, so that it can implement a series of tasks and strategies for tourism experience management, and provide guidance for the management of tourism industry.
(2) The overall development level of the ecological environment system in Nagasaki Prefecture is in a declining stage. Therefore, Nagasaki Prefecture should pay more attention to the protection of the ecological environment while also monitoring the development of the tourism economy. To this end, first, it should raise public awareness of as well as provide education on environmental protection. Second, the prefecture needs to improve its control over the discharge of waste gas, waste, and wastewater from enterprises, and promote the use of clean energy. Third, it should strengthen the supervision and enforcement of environmental protection and improve relevant laws on environmental protection.
(3) Coordination between the development of tourism and the ecological environment in the prefecture is good, which is the key to sustainable development of the tourism economic system and the ecological environment system. Therefore, the prefecture should vigorously develop eco-tourism through financial support as well as preferential policies, tax breaks and exemption policies for the industry. However, the prefecture needs to implement strict environmental protection measures to achieve coordinated and sustainable development of tourism and the ecological environment.

## 6. Conclusions

This research involved constructing a PSR model of the direct coupling and coordination extremes between the tourism economy and the ecological environment for Nagasaki Prefecture, based on a pressure-state-correspondence framework and the characteristics of the prefecture’s ecological environment. The coupling degree model and the coupling coordination degree model were introduced to measure the comprehensive development index of the tourism economic system and the ecological environment system in the prefecture. The state of coupled and coordinated development of the tourism economy and the ecosystem was then analyzed and evolving trends were noted. The results of this paper on Nagasaki Prefecture show that:

(1) Nagasaki Prefecture’s tourism economic development index showed an increasing trend from 2010 to 2019, but the prefecture’s ecological environment development index showed a fluctuating decline;
(2) From 2010 to 2019, the coupling degree between the tourism economic system and the ecological environment system of Nagasaki Prefecture is mainly in the antagonistic stage, and the coupling degree between the two needs to be improved;
(3) From 2010 to 2019, the coupling coordination degree of the tourism economy and ecological environment system of Nagasaki Prefecture changed from moderate disorder to light disorder.

Therefore, tourism planners and government organizations in Nagasaki Prefecture should pay more attention to the impact of tourism revenue and focus on optimizing the structure of tourism when designing tourism development policies. In addition, it is particularly important for Nagasaki Prefecture, while attaching importance to the development of its tourism economy, to pay more attention to protection of the ecological environment in pursuit of a higher quality ecological environment.

It is worth noting that through the case analysis of Nagasaki Prefecture, Japan, this study not only confirms that the combination of CCDM and PSR can help local governments better solve complex coupling relationships, but can also help in formulating sustainable development strategies for the tourism economy and ecological environment.

This paper covers the period from 2010 to 2019, and all statistical data were obtained from official Japanese websites. As a continuous series of some indicator data were not obtainable, this research only selected data for 2010, 2013, 2016 and 2019, which led to certain limitations in the research results. If the research timeline could be extended and the spatial dimension analysis were to include data from all cities in Nagasaki Prefecture, the research would more clearly reflect the evolution of progress in the coupling and coordination of the tourism industry and the ecological environment system in the prefecture. In future follow-up studies, researchers will need to conduct in-depth investigations of typical variations across regions, and pay particular attention to the time–space comparison of different regions. In this way, researchers can analyze not only the development level of tourism economy and ecological environment more accurately and comprehensively, but also explore ways to realize the coordinated development of industry and environment, and enhance the pertinence of research.

## Figures and Tables

**Table 1:** Index system used for evaluation of the relationship between tourism economy and eco-environment.

Subsystem	First-Class Index	Second-Class Index	Unit
Tourism economy	Economic benefit	Total tourism revenue	Billion yen/year
		Proportion of total tourism revenue in the tertiary industry	%
		Total tourism revenue as a proportion of GDP	%
	Scale of development	Total number of tourists received	Ten thousand people
		Total number of foreign tourists received	Ten thousand people
		Total number of tourist reception accounts as a percentage of the permanent population	%
	Tourism supply	Number of main tourist facilities	Places
		Number of places of interest	Places
		Number of accommodation facilities	Places
Eco-environment	Pressure	Discharge of domestic waste per person per day	g/person·day
		Water pollutant discharge	Thousand tons/year
		Waste discharge	Thousand tons/year
		Garbage discharged per person per day	g/person/day
	State	Dam impoundment rate	%
		Forestry rate	%
		Park green area per capita	Hectares
	Response	Waste treatment rate	%
		Population penetration rate of sewage treatment	%
		Waste recycling rate	%

**Table 2:** Classification of coupling coordination degree.

Category	Coupling Coordination Degree	Subclass
Disorder (Zone of unacceptable)	0.00–0.09	Extreme disorder
	0.10–0.19	Serious disorder
	0.20–0.29	Moderate disorder
	0.30–0.39	Light disorder
Transition (Zone of reluctantly accept)	0.40–0.49	Near disorder
	0.50–0.59	Poorly coordinated
Coordination (Zone of tolerance)	0.60–0.69	Primary coordination
	0.70–0.79	Medium coordination
	0.80–0.89	Good coordination
	0.90–1.00	High coordination

**Table 3:** Coupling degree and coordination degree between tourism economy and ecological environment in Nagasaki Prefecture.

	P_TE_	P_EE_	C	T	D
2010	0.084	0.245	0.436	0.164	0.268
2013	0.191	0.298	0.488	0.245	0.345
2016	0.301	0.083	0.412	0.192	0.282
2019	0.424	0.133	0.426	0.279	0.345

**Table 4:** Coupling measurement standard [56].

C	Stage
0 < C ≤ 0.3	Low-level coupling
0.3 < C ≤ 0.5	Antagonistic stage
0.5 < C ≤ 0.8	Run-in stage
0.8 < C ≤ 1	High level of coupling

## Data Availability

All sources have been provided in the paper.
